# Hospital Choirs

**Published:** 1890-11-01

**Authors:** 


### Passing Topics.

### HOSPITAL CHOIRS.

In these days much that is graceful and refined enters into hospital life. Science is no longer disdainful of the help wrought by sympathy in winning sick people back to health, and a period of moral as well as of material sanitation has set in. It is not enough that a hospital should be scrupulously clean; it must be purged of gloom, it must be redolent of flowers, it must bear witness at every turn of those high principles which of late have affected hospital work so largely and have raised the care and tendance of the sick to the level of a fine art.

The picture presented by the interior of any well-appointed and capably-administered hospital is eminently one upon which the eye may rest with satisfaction, and nothing more surprises the stranger than the absence from the reality of of many of those more obtrusive features of pain and sorrow with which the imaginary scene has been so lavishly furnished. True, the pain and the sorrow are there, but they do not intrude themselves upon the senses. There is, and it is a very important fact, no intercommunication of suffering, such as inevitably arises when sick people are herded together under less favourable circumstances, and illness assumes the character of a pestilence.

At this time of year, when winter impends, it ought not to be difficult to bespeak favourable attention for a suggestion to make a seasonable addition to the beneficent influences at work by the organisation on the part of each institution of a choir from the material ready to hand within its walls. Light, colour, and fragrance we have already—may not sweet sounds be enlisted too? We can conceive of few things more likely to bring delight and advantage both to those who labour and those who are laboured for, than an ever-present means to summon music to their solace. Hospital entertainments and concerts have now a recognised value, and we are glad to observe as we write the announcement of a Musical Society which purposes to visit hospitals, but general gatherings cannot occur daily; while a large proportion, both of patients and nurses, must always be unable to share in them. The hospital choir would be composed, doubtless, in chief part of nurses. It would be available as a whole or in detachments, and might be made the means of imparting a beauty and a finish to many ordinary and every-day functions, whether religious or secular.

We all know what intense gratification is afforded by the custom followed in some institutions of singing carols in the wards at Christmas time. Just elaborate that idea but a little and we should get all we want. There need be no fear that the pleasure would grow stale. Even if we forget that music yields perennial delights and bears repetition, we may remember that to the ever-changing occupants of the wards the charm of novelty would be always present. To the nurses themselves, the accomplishment could not fail to be a source of pleasant and healthful recreation, and if stimulus were needed to prevent interest from flagging, it would be easy to arrange for an exchange of visits upon rare occasions between the representatives of different hospitals. Friendly emulation would induce efficiency and add zest to the enjoyment, while if anything further is needed to commend the proposal, it is that its adoption would involve little or no expense, and need not therefore beget that terrible penultimus—an appeal for funds.

